# Zirconia implants interfere with the evaluation of peri-implant bone defects in cone beam computed tomography (CBCT) images even with artifact reduction, a pilot study

**DOI:** 10.1259/dmfr.20230252

**Published:** 2023-09-04

**Authors:** Niina Kuusisto, Faleh Abushahba, Stina Syrjänen, Sisko Huumonen, Pekka Vallittu, Timo Närhi

**Affiliations:** 1 Department of Oral Pathology and Radiology, Institute of Dentistry, University of Turku, Turku, Finland; 2 Department of Radiology, Päijät-Häme Central Hospital, Lahti, Finland; 3 Department of Prosthetic Dentistry and Stomatognathic Physiology, Institute of Dentistry, University of Turku, Turku, Finland; 4 Institute of Dentistry, University of Eastern Finland, Kuopio, Finland; 5 Diagnostic Imaging Center, Kuopio University Hospital, Kuopio, Finland; 6 Department of Biomaterials Science and Turku Clinical Biomaterials Centre – TCBC, Institute of Dentistry, University of Turku, Turku, Finland; 7 Welfare Division, City of Turku, Turku, Finland

**Keywords:** peri-implantitis, artifact, cone beam computed tomography

## Abstract

**Objectives:**

Three-dimensional cone beam computed tomography (CBCT) imaging can be considered, especially in patients with complicated peri-implantitis (PI). Artifacts induced by dense materials are the drawback of CBCT imaging and the peri-implant bone condition may not be assessed reliably because the artifacts are present in the same area. This pilot study investigates the performance of the artifact reduction algorithm (ARA) of the Planmeca Viso G7 CBCT device (Planmeca, Helsinki, Finland) with three different implant materials and imaging parameters.

**Methods:**

Three pairs of dental implants consisting of titanium, zirconia, and fiber reinforced composite (FRC) were set into a pig mandible. A vertical defect simulating peri-implantitis bone loss was made on the buccal side of one of each implant. The defect was identified and measured by two observers and compared to the actual dimensions. In addition, the bone structure and the marginal cortex visibility between the implants were estimated visually.

**Results:**

The bone defect and its dimensions with the zirconia implant could not be identified in any image with or without the metal artifact reduction algorithm. The bone defect of titanium and FRC implants were identified with all three imaging parameters or even without ARA. The interobserver agreement between the two observers was almost perfect for all categories analyzed.

**Conclusion:**

Peri-implantitis defect of the zirconia implant and the peri-implant bone structure of the zirconia implants cannot be recognized reliably with any ARA levels, or any imaging parameters used with the Planmeca Viso G7. The need for ARA when imaging the peri-implant bone condition of the titanium and FRC implants may be unnecessary.

## Introduction

Peri-implantitis (PI) is an irreversible infectious disease around a dental implant which can eventually cause implant failure.^1^ PI is defined by the 2017 World Workshop on the Classification of Periodontal and Peri-Implant Diseases and Conditions as a pathological inflammation of peri-implant tissues with progressive loss of supporting bone detected radiographically.^2^ Symptoms are typically bleeding on probing, bone destruction, suppuration on probing, and swollen tissues.^3^ The prevalence of peri-implantitis is estimated to be more than 20%.^4,5^

To prevent implant failure, the clinical recognition of mucositis, which is the early stage of peri-implantitis, is essential to accomplish early enough. Intraoral images are recommended as the standard imaging method for follow-up of peri-implant conditions.^6,7^ However, due to the superimposition of the implant and the anatomical structures, two-dimensional images lack information on buccal and lingual/palatal vertical bone loss. Also, superimposition in intraoral radiographs can cause an underestimation of the bone defect dimensions.^8,9^ Three-dimensional cone beam computed tomography (CBCT) imaging offers reliable visualization and dimensions in three cross-sectional views. CBCT can be considered especially in complicated PI cases.^10^ The drawback of CBCT imaging is artifacts caused by dense materials, which have a high atomic number, such as implants. In CBCT images, artifacts are seen in the image as bright streaks, darkening areas and loss of gray values between metallic or dense objects.^11–14^ The peri-implant bone condition may not be assessed reliably in CBCT images since the artifacts are in the same area. Due to artifacts, CBCT is not recommended as a primary or routine imaging method when evaluating peri-implant marginal bone.^10^

Multiple metal artifact reduction algorithms have been developed for the latest CBCT devices. Although most recent studies recommend metal artifact reduction algorithms,^15,16^ the results, study setups, and used dental materials vary, so a unanimous recommendation for their use does not exist.

Titanium and its alloys have long been a successful dental implant materials, but they cause artifacts, especially when multiple titanium implants are present.^11,13,14^ In addition to titanium implants, *in vitro* and *in vivo* studies show that zirconia is a valuable implant material.^17^ However, zirconia implants are studied to create even more detrimental artifacts in the CBCT images than titanium.^18–20^ Recently, composite-based materials, such as fiber reinforced composite (FRC), have been investigated as potential dental implant materials. So far, it seems that composite-based materials do not cause any detrimental effects in the CBCT images.^21^

This pilot study investigates the performance of the artifact reduction algorithm (ARA) of the Planmeca Viso G7 CBCT unit (Planmeca, Helsinki, Finland) with three pairs of dental implants consisting of titanium, zirconia, and FRC, set into a pig mandible. One implant of each material has a vertical defect simulating peri-implantitis related bone loss. The functionality of the metal artifact reduction algorithm is analyzed by recognizing the defect and measuring the defect size compared to the actual dimensions. In addition, the bone structure and the marginal cortex visibility between the implants are estimated visually. The aim is to determine appropriate ARA levels in post-operative CBCT images for titanium and zirconia as well as for FRC as a potential dental implant material.

## Methods and materials

One pair of dental implants of each material: titanium (Straumann^®^ Bone Level Implant, Straumann Holding AG, Basel Switzerland), zirconia (Straumann^®^ PURE Ceramic Implant Monotype, Straumann Holding, AG, Basel Switzerland) and handmade FRC (University of Turku, Turku, Finland) were included in this study. The implants, size 3.3 × 10 mm, were placed side-by-side at a 5 mm distance from each other into a defrosted pig mandible using standard implant placement protocols of the manufacturer (Straumann Holding AG). Two glass fiber reinforced composite replicas of the used Straumann’s zirconia implant were prepared by making a translucent polyvinyl siloxane (Exaclear, GC, Tokyo, Japan) split mold of the zirconia implant. The mold was filled with two pieces of light-curing dimethacrylate resin impregnated continuous unidirectional E-glass fiber rovings (everStick C&B, Stick Tech-GC Group, Turku, Finland) and discontinuous E-glass fiber reinforced composite (everX Flow, bulk shade, GC, Tokyo, Japan). Composition of E-glass was SiO_2_ 54 wt%, Al_2_O_3_ 14 wt%, CaO+MgO 22 wt%, B_2_O_3_ 10 wt% and Na_2_O+K_2_O less than 2 wt%. The split-mold was closed, and the composites were light-cured by hand-held dental light curing unit for 40 s from two sides of the mold. The composite implant replicas were removed from the mold and finished.

**Figure 1.**
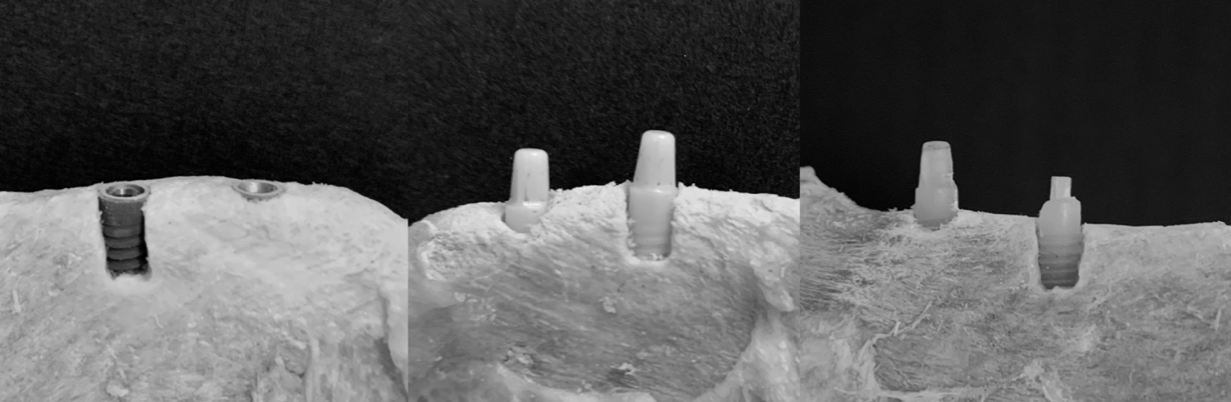
From left: titanium, zirconia and FRC implants placed into the mandibula. FRC, fiber reinforced composite

Zirconia implants were placed on the opposite side of the mandible than the titanium and FRC implants. The defect was created on the buccal side of one titanium, zirconia and FRC implant. The defect size was equal in each implant, 3 mm in width, 5 mm in height and 1 mm in depth on the caudal side of the defect. The other implant of each material was placed without a defect (Figure 1.). The soft tissues of the pig mandible were peeled off before implant placements.

The implants were imaged with CBCT (Planmeca Viso G7, Helsinki, Finland) with three different parameter options as follows: (1) whole mandible FOV 22 × 12 cm, 100 kV, 12 mA, 5 s with a resolution of 300 µm; (2) the implant pairs of each material separately, FOV 5 × 5 cm, 90 kV, 14 mA, 4.5 s with a resolution of 150 µm and (3) the implant pairs of each material separately, FOV 5 × 5 cm, 100 kV, 12 mA, 12.8 s with a resolution of 75 µm. All metal artifact reduction (ARA) levels (1–3) were performed with all imaging parameter options. Two observers with different backgrounds, an oral and maxillofacial radiologist (Observer 1) and a Bachelor of Dental Sciences (Observer 2), analyzed the images. The observers were calibrated how to measure the defect and to analyze the images under the same conditions. To define the defect size, it had to be recognizable in all three views (axial, sagittal, coronal) in the CBCT image. Both observers measured the defect size with the measuring tool in Romexis Viewer (Romexis Viewer 6, Planmeca, Helsinki, Finland). The defect’s height and depth were measured in the coronal view of the CBCT image, and the defect’s width was measured in the axial view of the CBCT image. The bone structure visibility, the marginal cortex between the implant pairs, and the spiral structure of the implants were analyzed visually (yes/no). The bone structure was required to be seen smooth between the implants, and the marginal cortex was required to be seen intact between the implants. The spiral structure of the implant was required to be seen in detail. CBCT images were assessed on an Eizo RadiForce GX340 diagnostic monitor with three megapixels and a resolution of 1536 × 2048 (Eizo Nanao Corporation, Ishikawa, Japan).

### Statistical analyses

Interobserver agreement was calculated using the interobserver class correlation coefficient test (ICC-test, parallel model with two-way random effects, absolute agreement type). SPSS 29 (IBM, NY) software package was used. The results of the ICC-test were classified as poor 0.20–0.40: moderate >0.40–0.60; substantial >0.60–0.80; almost perfect >0.80–1.00.

## Results

**Figure 2.**
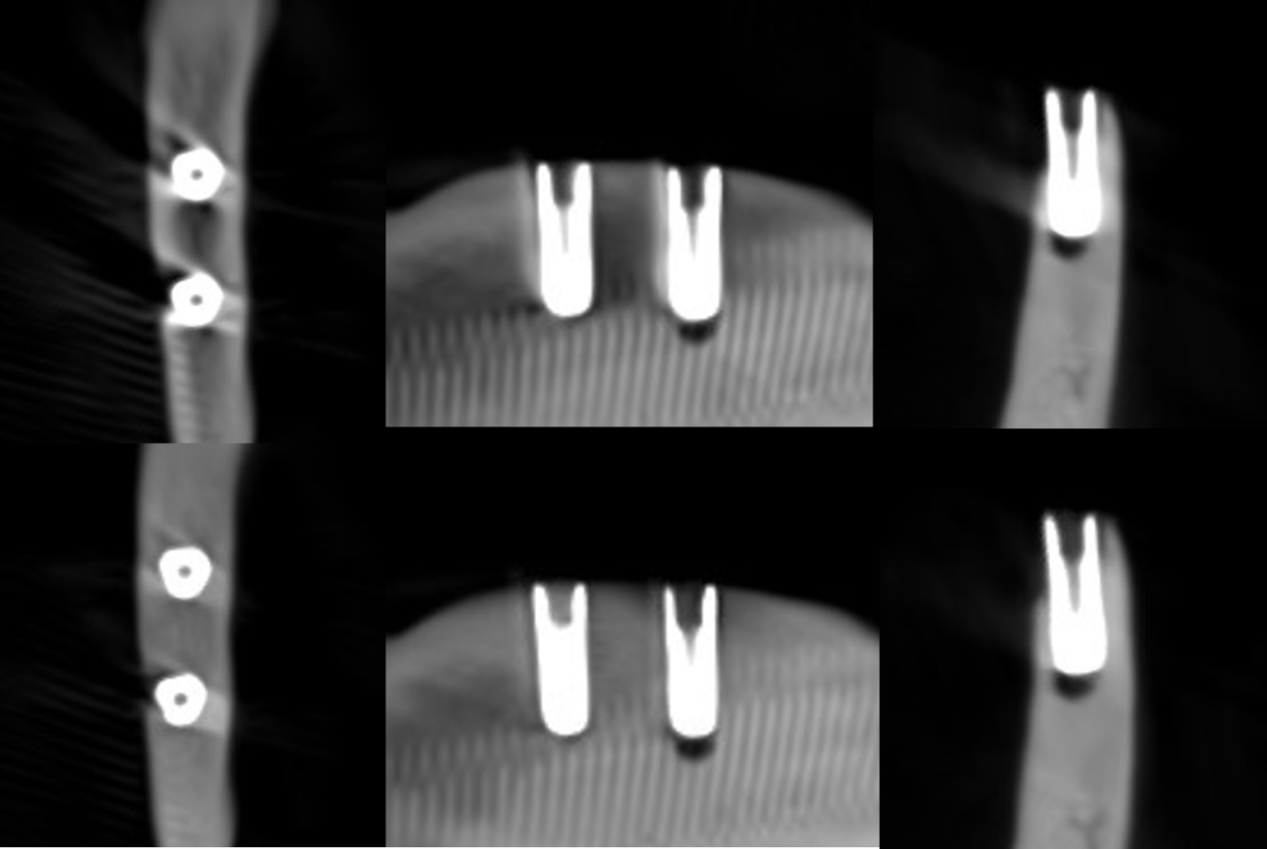
Axial, sagittal, and coronal slices of the CBCT image of the titanium implants with the imaging parameters 1. Above no ARA and below ARA 3. The defect is present in axial and coronal slices, and the bone structure between the implants is better seen with ARA 3. ARA, artifact reduction algorithm

**Figure 3.**
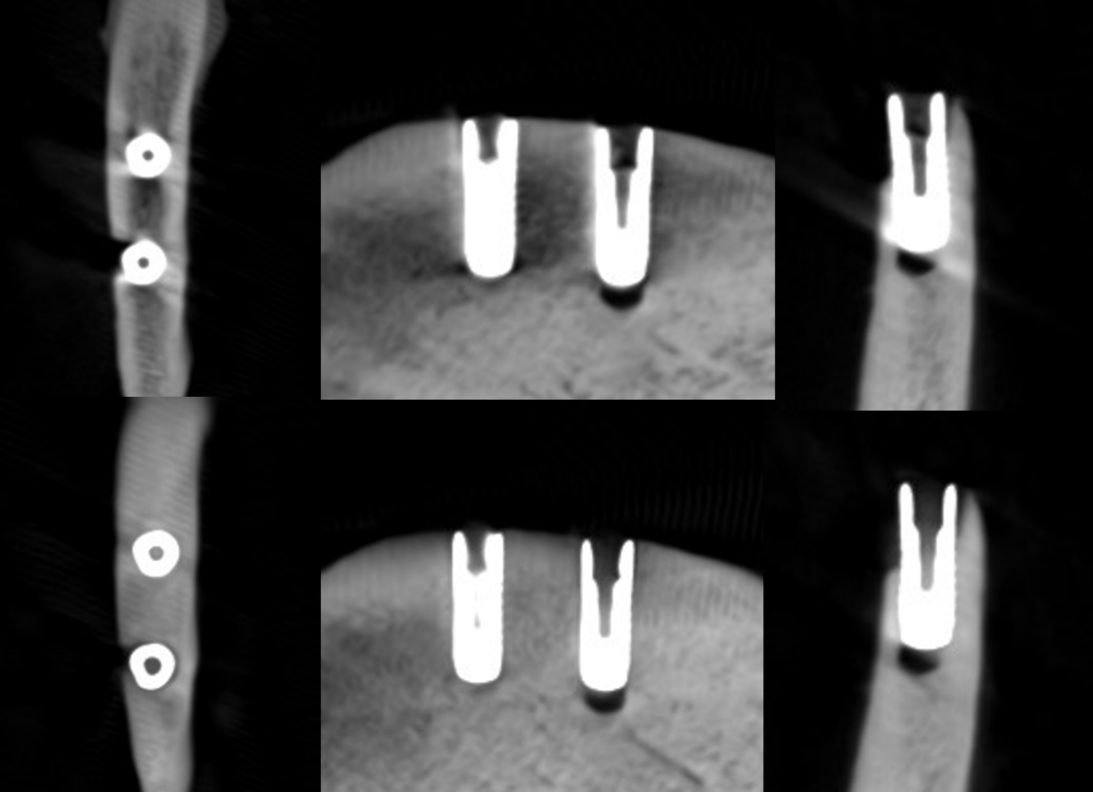
Axial, sagittal, and coronal slices of the CBCT image of the titanium implants with the imaging parameters 2. Above no ARA and below ARA 3. The defect is present and the bone structure between the implants is smoother with ARA 3. ARA, artifact reduction algorithm; CBCT, cone beam CT.

**Figure 4.**
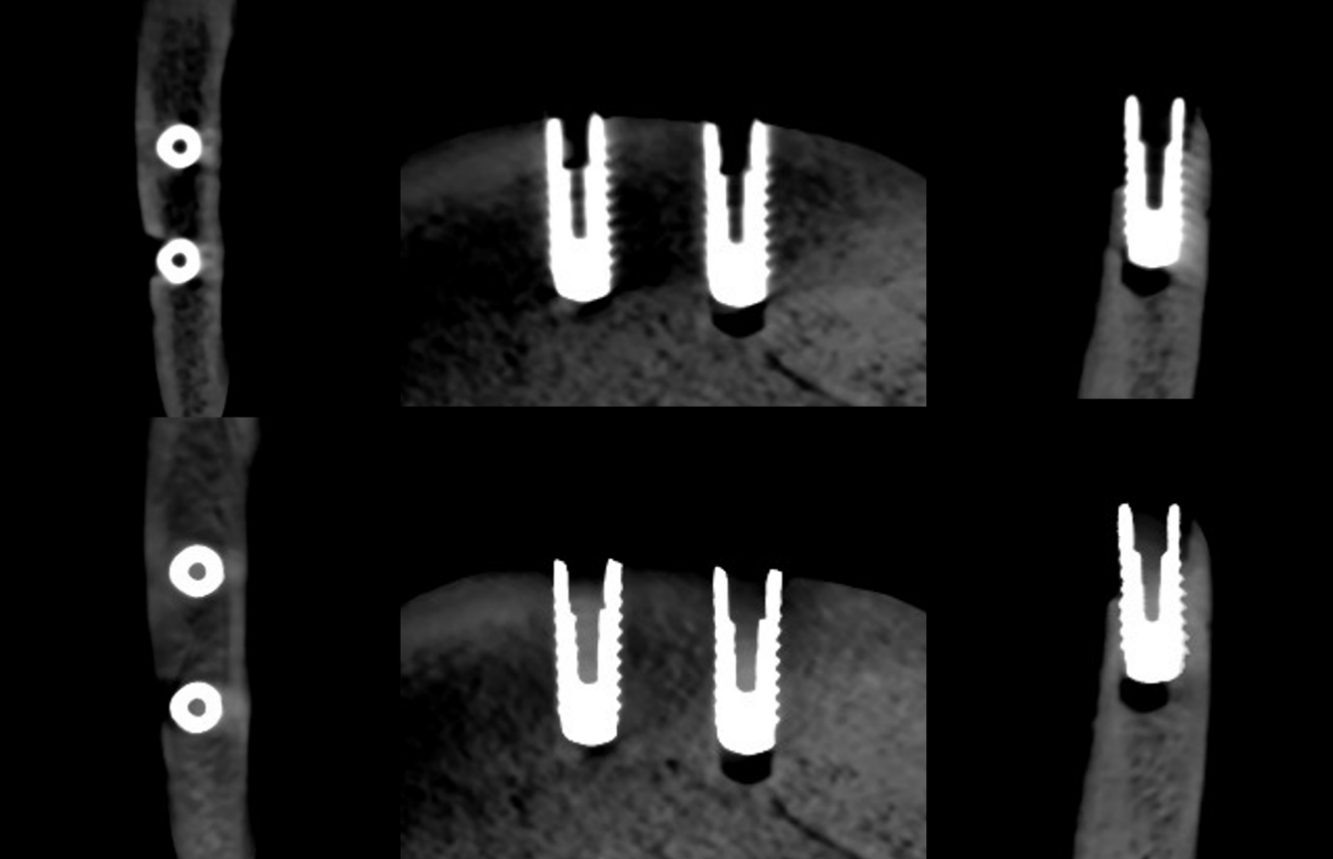
Axial, sagittal, and coronal slices of the CBCT image of the titanium implants with the imaging parameters 3. Above no ARA and below ARA 3. The defect is present in the images and the bone structure between the implants is smooth with ARA 3. The spiral structure is seen in detail. ARA, artifact reduction algorithm; CBCT, cone beam CT.

**Figure 5.**
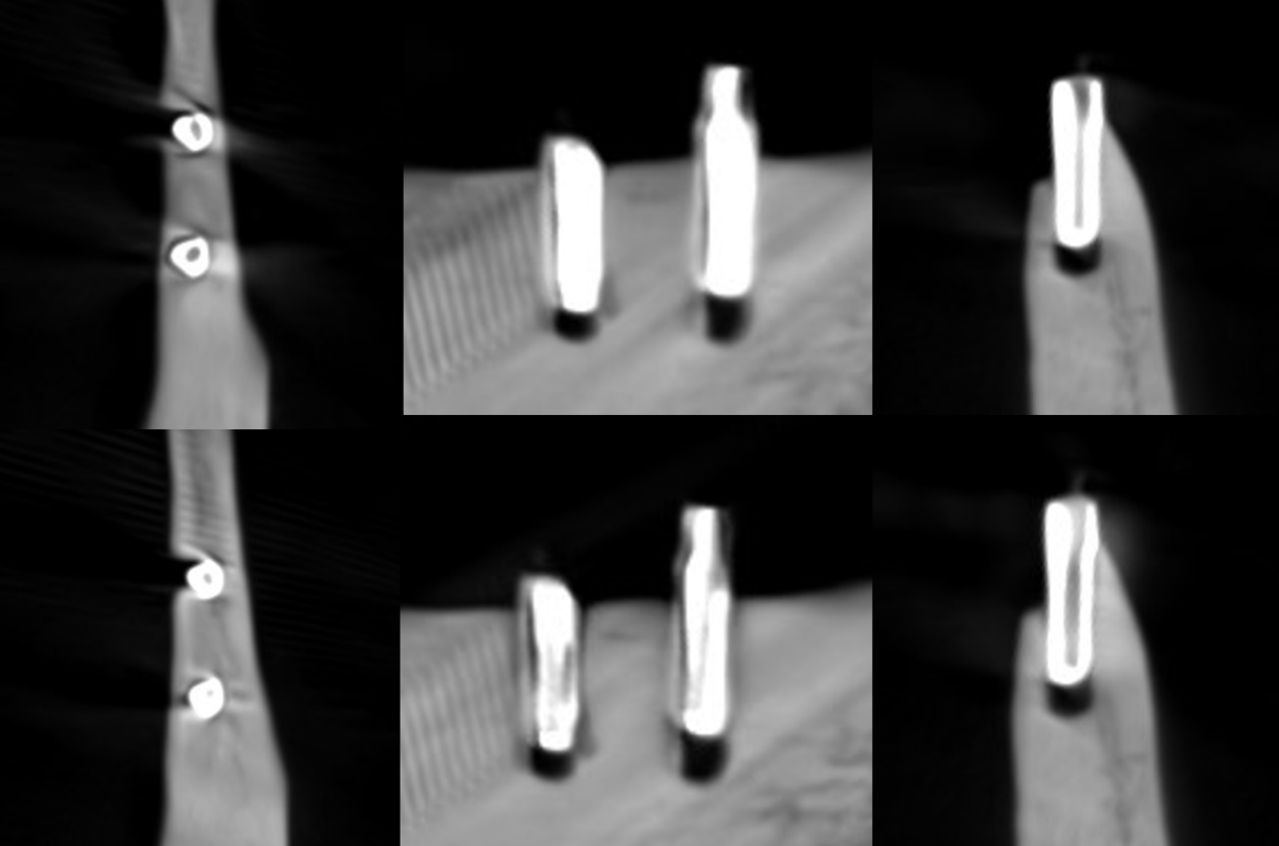
Axial, sagittal, and coronal slices of the CBCT image of the FRC implants with the imaging parameters 1. Above no ARA and below ARA 3. The defect is present in axial and coronal slices and the bone structure between the implants is seen with or without ARA. ARA, artifact reduction algorithm; CBCT, cone beam CT; FRC, fiber reinforced composite.

**Figure 6.**
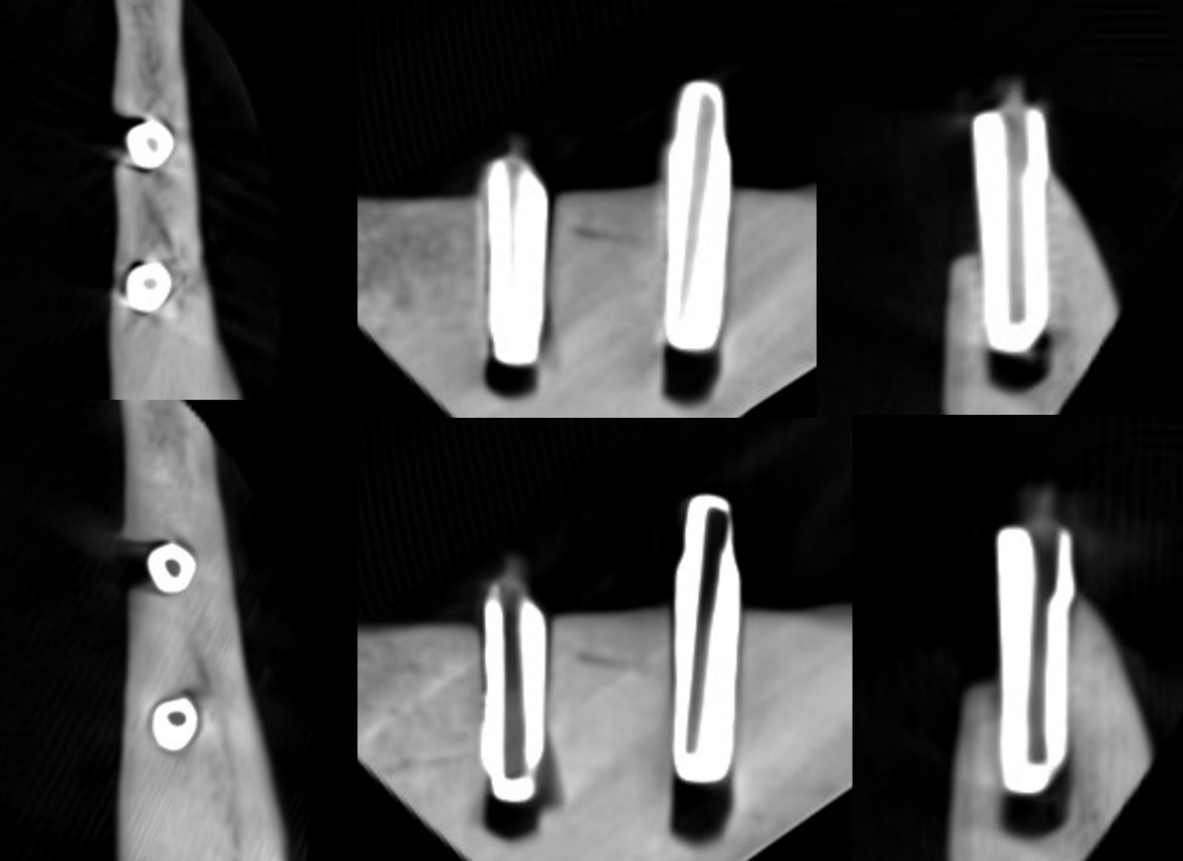
Axial, sagittal, and coronal slices of the CBCT image of the FRC implants with the imaging parameters 2. Above no ARA and below ARA 3. The defect is present and the bone structure between the implants is smooth with or without ARA. ARA, artifact reduction algorithm; CBCT, cone beam CT; FRC, fiber reinforced composite.

**Figure 7.**
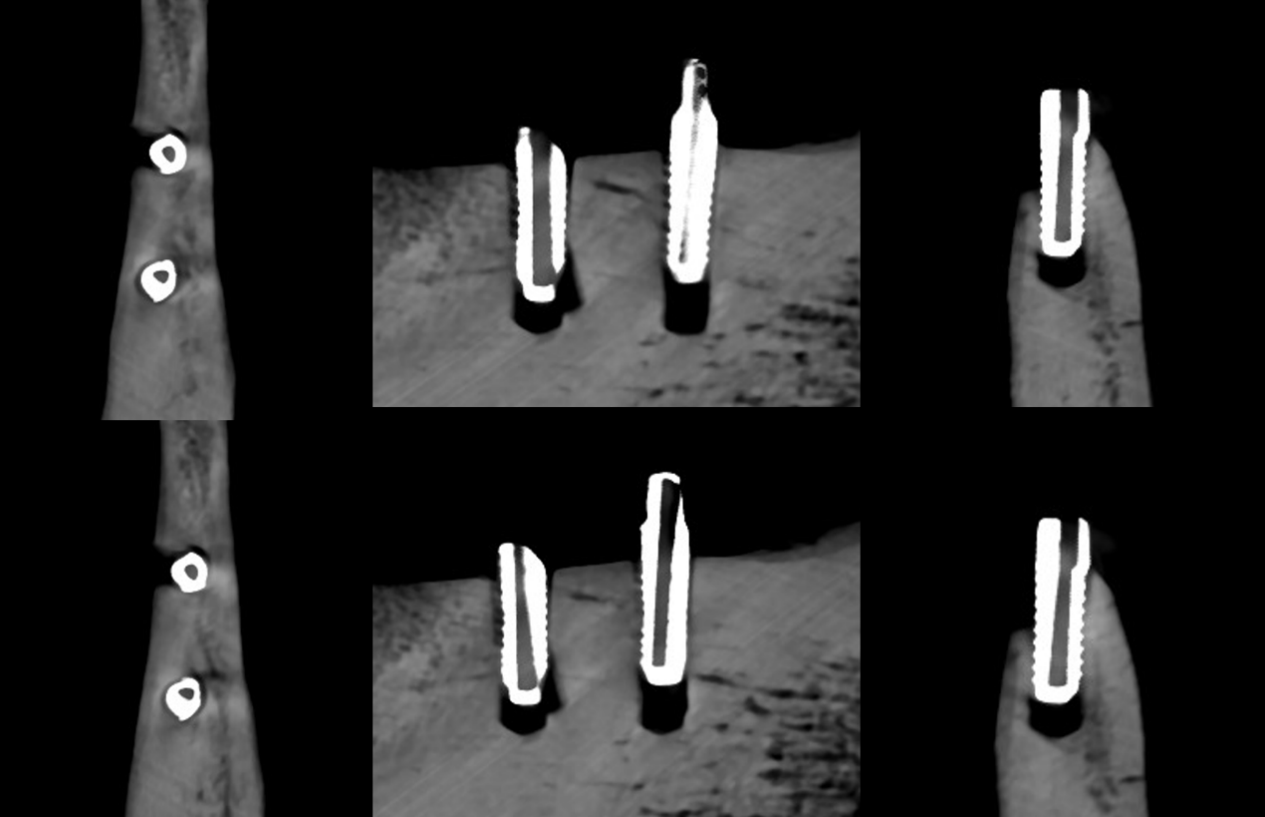
Axial, sagittal, and coronal slices of the CBCT image of the FRC implants with the imaging parameters 3. Above no ARA and below ARA 3. The defect is present in images and the bone structure between the implants is smooth with or without ARA. The spiral structure of the implants is seen in detail. ARA, artifact reduction algorithm; CBCT, cone beam CT; FRC, fiber reinforced composite.

**Figure 8.**
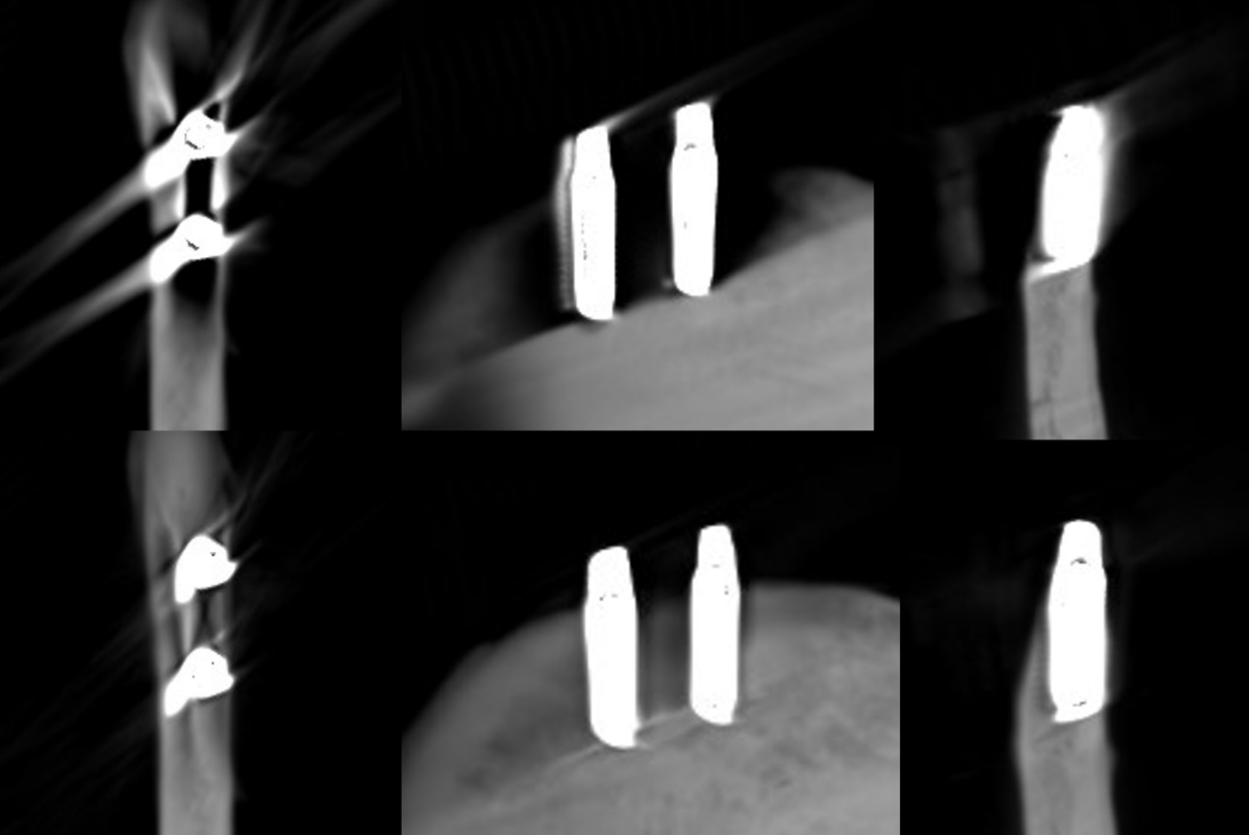
Axial, sagittal, and coronal slices of the CBCT image of the zirconia implants with the imaging parameters 1. Above no ARA and below ARA 3. The defect cannot be seen and the bone structure between the implants is not smooth in even with ARA 3. ARA, artifact reduction algorithm; CBCT, cone beam CT.

**Figure 9.**
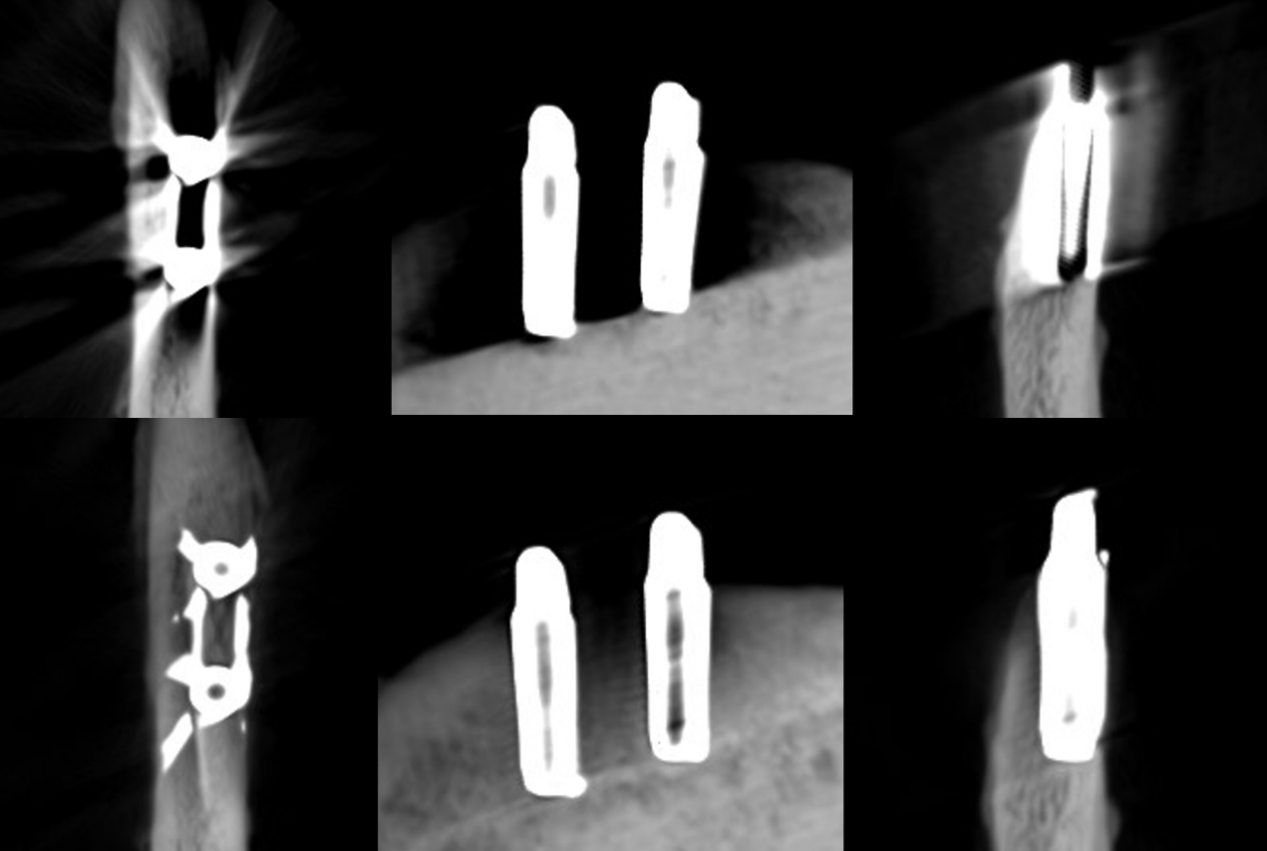
Axial, sagittal, and coronal slices of the CBCT image of the zirconia implants with the imaging parameters 2. Above no ARA and below ARA 3. The defect cannot be seen and the bone structure between the implants is not smooth. ARA, artifact reduction algorithm; CBCT, cone beam CT.

**Figure 10.**
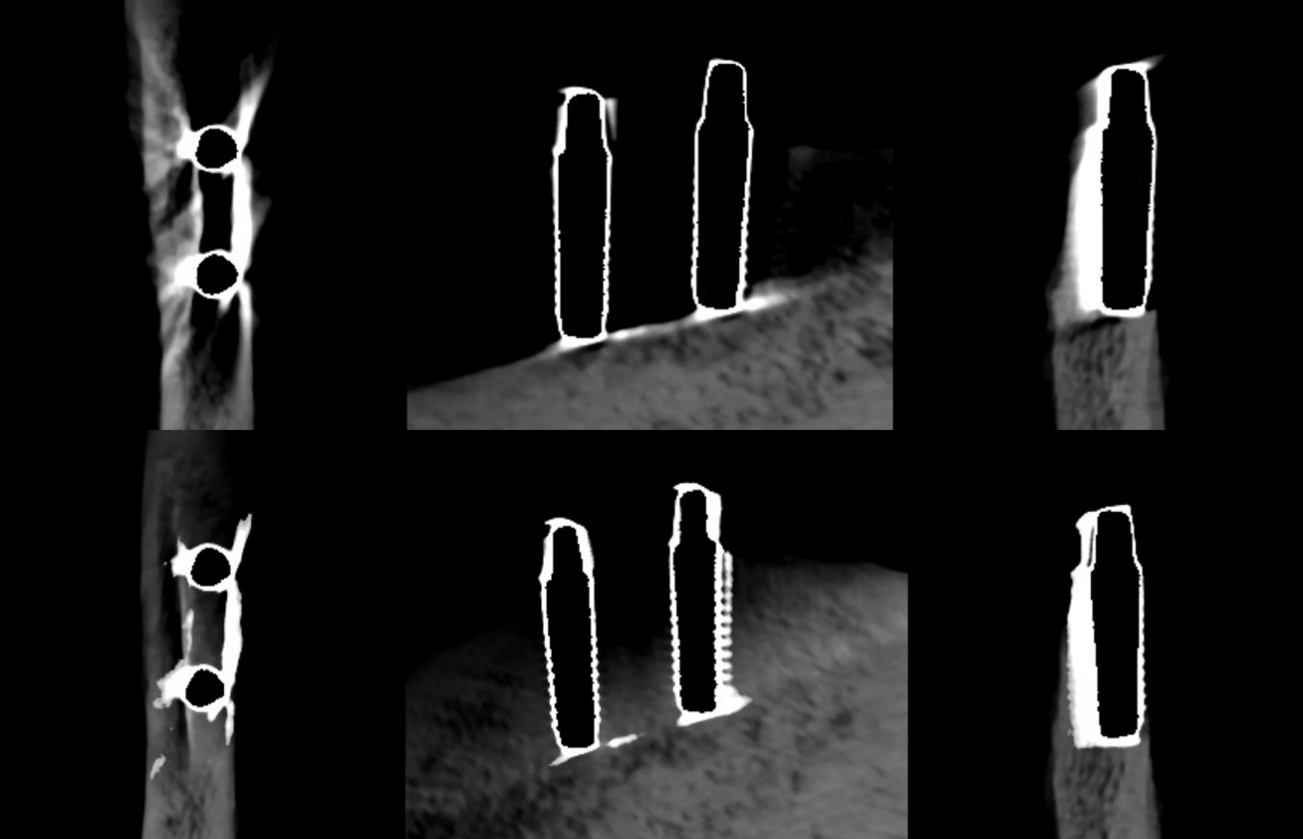
Axial, sagittal, and coronal slices of the CBCT image of the zirconia implants with the imaging parameters 3. Above no ARA and below ARA 3. The defect cannot be seen and the bone structure between the implants is not smooth. In addition, the zirconia implants are seen centrally radiolucent with a radiopaque rim with or without ARA. ARA, artifact reduction algorithm; CBCT, cone beam CT.

The buccal bone defect of titanium (Figures 2–4) and FRC (Figures 5–7) implants were identified in all images and with all levels of ARA. The buccal bone defect and its dimensions with the zirconia implant could not be recognized in any image with or without the metal ARA (Figures 8–10). The interobserver agreement between the two observers was almost perfect for all categories analyzed. Both observers made their measurements only once. Thus, to calculate the interobserver agreements with the ICC-test, the measured height, width, and depth for each material with or without ARA observed by Observer 1 was compared to those measured by Observer 2.

The results of the measurements of the defects by both observers and their interobserver agreement are shown in detail in Tables 1–3.

**Table 1.** The defect size with imaging parameters 1^*a*^

Defect size	Height mm		Width mm		Depth mm		ICC (95% CI)
**Observer**	**1**	**2**	**1**	**2**	**1**	**2**	
*Zirconia*							
No ARA	0	0	0	0	0	0	NA
ARA 1	0	0	0	0	0	0	NA
ARA 2	0	0	0	0	0	0	NA
ARA 3	0	0	0	0	0	0	NA
*Titanium*							
No ARA	5.4	5.4	3	3.3	1	1.2	0.998 (0.923–1.000)
ARA 1	6	5.4	2.5	2.1	1.2	0.9	0.991 (0.195–1.000)
ARA 2	5.1	5.1	2.1	1.8	0.9	0.9	0.998 (0.975–1.000)
ARA 3	5.3	5.1	2.4	1.8	1.2	0.6	0.987 (0.210–1.000)
*FRC*							
No ARA	5	5.9	2.4	2.4	1.2	1.8	0.977 (0.446–0.999)
ARA 1	5	5.3	2.1	2.7	1.2	1.5	0.988 (0.183–1.000)
ARA 2	5.3	5.9	2.3	2.1	1.2	1.2	0.994 (0.857–1.000)
ARA 3	5.3	6.2	2.2	1.8	1.2	1.2	0.986 (0.588–1.000)

ARA, artifact reduction algorithm; FRC, fiber reinforced composite; ICC, intraclass correlation coefficient;NA, not applicable;Observer 1, oral and maxillofacial radiologist; Observer 2, bachelor of dental sciences.

a Whole mandible, FOV 22 × 12 cm, 100 kV, 12 mA, 5 s with a resolution of 300 µm

**Table 2.** The defect size with imaging parameters 2^*a*^

Defect size	Height mm		Width mm		Depth mm		ICC (95% CI)
**Observer**	**1**	**2**	**1**	**2**	**1**	**2**	
*Zirconia*							
No ARA	0	0	0	0	0	0	NA
ARA 1	0	0	0	0	0	0	NA
ARA 2	0	0	0	0	0	0	NA
ARA 3	0	0	0	0	0	0	NA
*Titanium*							
No ARA	5.4	5.6	2.7	2.7	0.75	0.8	0.999 (0.988–1.000)
ARA 1	5.1	5.6	2.1	2.1	0.75	0.8	0.996 (0.937–1.000)
ARA 2	5.1	5.3	2.3	2	0.75	0.6	0.998 (0.947–1.000)
ARA 3	5.1	5.3	2.3	2	0.75	0.8	0.998 (0.916–1.000)
*FRC*							
No ARA	5.3	6.1	2.7	2.1	1	1.2	0.984 (0.420–1.000)
ARA 1	5.3	5.9	2.7	2.9	1	1.3	0.992 (0.441–1.000)
ARA 2	5.3	5.9	2.7	2.9	1	1.2	0.993 (0.626–1.000)
ARA 3	5.3	5.8	2.7	3	1	1.2	0.995 (0.990–0.998)

ARA, artifact reduction algorithm; FOV, field of view; FRC, fiber reinforced composite; ICC, intraclass correlation coefficient;NA, not applicable;Observer 1, oral and maxillofacial radiologist; Observer 2, bachelor of dental sciences.

a The implant pairs of each material separately, FOV 5 × 5 cm, 90 kV, 14 mA, 4.5 s with a resolution of 150 µm.

**Table 3.** The defect size with imaging parameters 3^*a*^

Defect size	Height mm		Width mm		Depth mm		ICC (95% CI)
*Observer*	**1**	**2**	**1**	**2**	**1**	**2**	
Zirconia							
No ARA	0	0	0	0	0	0	NA
ARA 1	0	0	0	0	0	0	NA
ARA 2	0	0	0	0	0	0	NA
ARA 3	0	0	0	0	0	0	NA
*Titanium*							
No ARA	5.1	5.4	2.5	3	0.8	0.5	0.993 (0.870–1.000)
ARA 1	5.1	5.5	2.7	2.9	0.8	0.5	0.996 (0.887–1.000)
ARA 2	5.1	5.3	2.7	2.9	0.8	0.5	0.997 (0.899–1.000)
ARA 3	5.1	5.4	2.7	2.6	0.8	0.5	0.997 (0.889–1.000)
*FRC*							
No ARA	5.4	6	2.7	1.6	1	1.3	0.976 (0.618–0.999)
ARA 1	5.3	6.1	2.6	2.8	1	1.3	0.988 (0.515–1.000)
ARA 2	5.4	5.4	2.6	1.9	1	1.2	0.991 (0.808–1.000)
ARA 3	5.4	5.4	2.7	2.9	1	1.2	0.999 (0.946–1.000)

ARA, artifact reduction algorithm; FOV, field of view; FRC, fiber reinforced composite; ICC, intraclass correlation coefficient;NA, not applicable;Observer 1, oral and maxillofacial radiologist; Observer 2, bachelor of dental sciences.

a The implant pairs of each material separately, FOV 5 × 5 cm, 100 kV, 12 mA, 12.8 s with a resolution of 75 µm.

With the imaging parameters 1, the defect size of the titanium implant between the observers varied with a range of 5.1–6 mm in height, 1.8–3.3 mm in width and 0.6–1.2 mm in depth. The dimensions of the defect, which differed from the real ones at most 0.5 mm, were achieved with ARA 2–3 or without ARA in height, without ARA in width and with or without ARA in depth by both observers (Table 1) (Figure 2). The defect size of the titanium implant with the imaging parameters 2 varied between the observers with a range of 5.1–5.6 mm in height, 2–2.7 mm in width and 0.6–0.8 mm in depth. The dimensions of the defect, which differed from the real ones at most 0.5 mm, were achieved with ARA 2–3 in height, without ARA in width and with or without ARA in depth by both observers (Table 2) (Figure 3). With the imaging parameters 3, the defect size of the titanium implant varied between the observers with a range of 5.1–5.5 mm in height, 2.5–3 mm in width and 0.5–0.8 mm in depth. The dimensions of the defect, which differed from the real ones at most 0.5 mm, were achieved with or without ARA in height, width, and depth (Table 3) (Figure 4).

The defect size of the FRC implant with the imaging parameters 1 varied between the observers with a range of 5.0–6.2 mm in height, 1.8–2.7 mm in width, and 1.2–1.8 mm in depth. The dimensions of the defect, which differed from the real ones at most 0.5 mm, were achieved with ARA 1 in height by both observers and ARA 1 by Observer 2 in width and ARA 1–3 in depth by both observers (Table 1) (Figure 5). With the imaging parameters 2, the defect size varied between the observers with a range of 5.3–6.1 mm in height, 2.1–3 mm in width and 1–1.3 mm in depth. The dimensions of the defect, which differed from the real ones at most 0.5 mm, were achieved with or without ARA in height by Observer 1, and ARA 1–3 in width by both observers and with or without ARA in depth by both observers (Table 2) (Figure 6f). With the imaging parameters 3, the defect size varied with a range of 5.3–6.1 mm in height, 1.6–2.9 mm in width and 1–1.3 mm in depth. The dimensions of the defect, which differed from the real ones at most 0.5 mm, were achieved with ARA 2–3 in height, ARA 1 and ARA 3 in width and with or without ARA in depth by both observers (Table 3) (Figure 7).

The bone structure or marginal cortex between the zirconia implants could not be identified as smooth in any images with or without ARA. The marginal cortex between titanium and FRC implants was able to identify in all images. The bone structure between titanium implants was smoother with ARA 2 and ARA 3 with all imaging parameters. Between FRC implants, the bone structure was easily seen with or without ARA and with all imaging parameters. The spiral structure of the titanium and FRC implants was recognized with imaging parameters 3 with or without ARA. Both observers agreed with the visibility of the implants’ marginal cortex, bone structure and spiral structure.

## Discussion

The present results showed that either the peri-implant bone structure or its defect with the zirconia implant cannot be recognized reliably with any ARA level or any imaging parameters used (Figures 8–10). The marginal cortex between titanium implants is clearly visible with or without ARA with all imaging parameters (Figures 2–4). The bone structure between the titanium implants is best seen using ARA 2 or 3. The PI defect of the titanium implant was reliably measured without ARA in all imaging parameters, but especially with imaging parameters 3 (Figures 2–4). The marginal cortex and the bone structure between the FRC implants were clearly visible with all imaging parameters at any ARA level (Figures 5–7). The PI defect of the FRC implant was reliably measured with varying levels of ARA in all imaging parameters. The spiral structure of the titanium and FRC implants is seen in detail with imaging parameters 3 with a resolution of 75 µm (Figure 7). The need for ARA when imaging the peri-implant bone condition of the titanium and FRC implants may be unnecessary, but in turn, ARA does not hamper the image quality either.

The success of dental implant is based on many factors, such as the bone quality, implant surface, overall health of the patient, and loading protocols.^22^ In the oral environment, dental implants are continuously exposed to microbes. To prevent peri-implant mucositis and PI, plaque control of the implant administered by professionals and the patient is an important prerequisite.^23^ Also, the position of the implant is crucial to the successful implant treatment. Malposition of the implant is clearly associated to PI.^24^

Intraoral radiographs are essential to assess and follow up the peri-implant bone condition.^6,7^ However, when assessing peri-implant bone status, various kinds of bone loss should be considered because these may be identified only with certain imaging methods. For example, in intraoral radiographs, horizontal and 1-wall mesiodistal vertical bone defects can be diagnosed in detail.^25^ In turn, studies show that CBCT imaging can be indicated for diagnosing fenestration, dehiscence and three-walled bone defects.^26,27^ The comparative animal study by Song and coworkers showed that CBCT provides essential information about peri-implant bone conditions that cannot be achieved by intraoral imaging.^28^ Also, Pelekos and coworkers showed that CBCT has better diagnostic accuracy in identifying peri-implant defects than digital periapical radiographs with *in vitro* model.^29^ CBCT imaging is recommended mainly for patients with complicated PI due to artifacts.^10^

FRCs, composed of a polymeric matrix and supporting fibers,^30^ are studied to meet all the mechanical requirements of the oral environment.^31–35^ The current application of FRCs are skull implants and bone substitutes,^36^ orbital floor and craniofacial bone reconstruction.^37^ Additional FRC applications are continuously investigated. FRC is not yet used as a dental implant material in clinical applications, although its properties have already been studied in laboratory conditions.^38,39^ However, preclinical investigations of FRC as a dental implant material are still needed. According to the results of this pilot study, non-metallic FRC does not cause artifacts in CBCT images and hence would be beneficial in areas with thin bone structures especially when post-operative CBCT imaging is needed. Titanium and its alloys are most popular materials used in dental implants and are shown to cause detrimental artifacts in the CBCT images.^11,13^ In terms of biomechanical and aesthetic properties zirconia is also considered successful implant material,^17,40^ but it is shown to create more detrimental artifacts in the CBCT images than titanium.^18–20^ This can be explained by the atomic numbers of the main material of the implant—40 for the element zirconium *vs* 22 for titanium.^20^ Material density (atomic weight of the material) affects the magnitude of the artifacts in CBCT images.^14^ In our study, the peri-implant bone defect of zirconia implants could not be assessed. This is contradictory to the results of the systematic review by Chagas and coworkers showing that the diagnostic accuracy of CBCT imaging of peri-implant bone defects was equivalent between titanium and zirconia implants.^41^

Metal ARAs are developed to improve image quality during data reconstruction.^42,43^ Currently, there are contradictory results of their use in different CBCT equipment and study sets. According to Vasconcelos and coworkers, metal ARAs reduce artifacts, but the performance is influenced by CBCT devices, different materials and FOV sizes.^44^ Fontenele and coworkers detected a vertical root fracture near the zirconia implant, concluding that the metal ARA activation is inefficient.^45^

In addition, increased tube current improves image quality in the presence of zirconia implants.^46^ Schriber and coworkers state that CBCT settings of low-, standard- or high dose affect only minimally the impact of artifacts.^47^ In addition, a recent study of Nascimento and coworkers found that the use of varied milliamperages and the artifact reduction tool of the OP300 Maxio CBCT unit did not affect the diagnosis of peri-implant dehiscences.^48^ As seen in Figure 10 of our pilot study, the imaging parameters 3 and the use of ARA did not improve the image quality, by contrast, with the parameters 3 zirconia implants are seen biased, centrally radiolucent with a radiopaque rim. The imaging parameters used with ARA should be investigated further especially when imaging zirconia implants.

Our *in vitro* study did not consider the radiation dose. However, the indication for CBCT imaging should always be justified because of the greater radiation dose compared to intraoral images. In addition, when choosing the imaging parameters to improve the image quality, the impact on radiation dose should always be considered.^49^ Image quality with low-dose protocols of different CBCT devices are important to investigate further with the indication of PI.

Our pilot study investigated two implants of each of the three materials. We found more image hampering artifacts with two zirconia implants than in studies with one implant.^28,47^ Multiple implants side-by-side cause more artifacts,^19^ hence the number of implants should be considered when comparing different study sets. This study had a low number of samples. We intended to compare the artifact reduction ARA functionality with three different implant materials, titanium, zirconia, and FRC and in post-operative CBCT images. The purpose of this pilot study is to bring out the different radiological features of these materials—disadvantages and benefits in post-operative CBCT images. Clinicians are supposed to be aware of the outcomes of the implant materials in the post-operative CBCT images. A novel titanium alloy composed of 15% zirconium and 85% titanium was recently introduced on implant markets (Roxolid^®^, Straumann, Basel, Switzerland). Our study did not include this material, but its radiologic appearance seems to reflect existing titanium alloy implants. However, this needs to be verified in future studies.

Only two observers with different backgrounds measured the defects. Although the results of the ICC test were excellent, further studies are needed with more samples and observers. In addition, the measuring tool used might impact the results, particularly the small dimensions required for accuracy. The height of the FRC implant was more challenging to measure because the implant structure was extended above the marginal bone, and the titanium implant was a bone level implant. The shape of the FRC and zirconia implants used in our study differed from the shape of the titanium implant. This might have a minor impact on the artifacts in the CBCT images. The implants were placed into the pig mandible, where the buccal and lingual bone is thin. Also, the soft tissues and other anatomical structures were absent in the present study, and the results might differ from the actual patients. The PI defect of this study was quite large, and smaller defects should be investigated further. To detect, classify and measure accurately the peri-implant bone defect is essential for appropriate treatments.^50^

## Conclusion

The results of this pilot study encourage carefulness when imaging zirconia implants with Planmeca Viso G7 CBCT device despite the metal artifact reduction options. PI defect of the zirconia implant and the peri-implant bone structure of the zirconia implants cannot be recognized reliably with any ARA level, or any imaging parameters used. The PI defect of the titanium implant was reliably measured without ARA in all imaging parameters. The PI defect of the FRC implant was reliably measured with varying levels of ARA in all imaging parameters. Hence, the need for ARA when imaging the peri-implant bone structure of the titanium and FRC implants may be unnecessary. More studies of metal ARAs with different phantoms, materials and varying imaging parameters are needed to achieve an ideal performance of the metal ARAs.
